# *N*-3-oxo-hexanoyl-homoserine lactone, a bacterial quorum sensing signal, enhances salt tolerance in Arabidopsis and wheat

**DOI:** 10.1186/s40529-020-00283-5

**Published:** 2020-03-10

**Authors:** Qian Zhao, Xiang-Yun Yang, Yao Li, Fang Liu, Xiang-Yu Cao, Zhen-Hua Jia, Shui-Shan Song

**Affiliations:** 1 Biology Institute, Hebei Academy of Sciences, 46th South Street of Friendship, Shijiazhuang, 050051 Hebei China; 2 College of Life Science, Hebei University, 180th East Road of Wusi, Baoding, China; 3 Hebei Engineering and Technology Center of Microbiological Control on Main Crop Disease, 46th South Street of Friendship, Shijiazhuang, China

**Keywords:** *N*-acyl-homoserine lactone, Quorum sensing, Salt tolerance, Arabidopsis, Wheat

## Abstract

**Background:**

*N*-acyl-homoserine lactones (AHLs) are the quorum sensing (QS) signal molecules to coordinate the collective behavior in a population in Gram-negative bacteria. Recent evidences demonstrate their roles in plant growth and defense responses.

**Results:**

In present study, we show that the treatment of plant roots with *N*-3-oxo-hexanoyl-homoserine lactone (3OC6-HSL), one molecule of AHLs family, resulted in enhanced salt tolerance in Arabidopsis and wheat. We found that the growth inhibition phenotype including root length, shoot length and fresh weight were significantly improved by 3OC6-HSL under salt stress condition. The physiological and biochemical analysis revealed that the contents of chlorophyll and proline were increased and the contents of MDA and Na^+^ and Na^+^/K^+^ ratios were decreased after 3OC6-HSL treatment in Arabidopsis and wheat under salt stress condition. Molecular analysis showed that 3OC6-HSL significantly upregulated the expression of salt-responsive genes including ABA-dependent osmotic stress responsive genes *COR15a, RD22, ADH* and *P5CS1,* ABA-independent gene *ERD1*, and ion-homeostasis regulation genes *SOS1, SOS2* and *SOS3* in Arabidopsis under salt stress condition.

**Conclusions:**

These results indicated that 3OC6-HSL enhanced plant salt tolerance and ABA-dependent and ABA-independent signal pathways and SOS signaling might be involved in the induction of salt resistance by 3OC6-HSL in plants. Our data provide a new insight into the plant–microbe inter-communication.

## Background

*N*-acyl-homoserine lactones (AHLs) are produced by many Gram-negative bacteria to communicate and coordinate the individual behavior in a cell density-dependent manner in bacterial populations. This signaling mechanism is called quorum sensing (QS). Bacterial QS is involved in many physiological processes, such as virulence, bioluminescence, sporulation, swarming, siderophore production, antibiotic biosynthesis, biofilm formation and plasmid conjugal transfer (Pearson et al. 1994; Parsek et al. 1999). In recent years, accumulating evidence indicates that AHLs also have an impact on plant cells (Mathesius et al. 2003; von Rad et al. 2008; Liu et al. 2012; Miao et al. 2012; Schikora et al. 2011; Shenk et al. 2014; Zhao et al. 2015, 2016). Plants change their gene expression, alter their protein profile, modify their development and enhance their defense responses if AHLs are present in their surroundings. Proteomic analysis has revealed that the expression of over 150 proteins were significant changed after treated with two different AHL types, *N*-3-oxo-dodecanoyl-homoserine lactone (3OC12-HSL) and *N*-3-oxo-hexadecanoyl-homoserine lactone (3OC16:1-HSL) in *Medicago truncatula* (Mathesius et al. 2003), and the level of 53 proteins were accumulated induced by *N*-3-oxo-octanoyl-homoserine lactone (3OC8-HSL) in Arabidopsis seedlings (Miao et al. 2012). These proteins were found to function in plant defense, stress response, flavonoid synthesis, plant hormone responses, the cytoskeleton, protein degradation and processing, energetics and metabolic activities (Mathesius et al. 2003; Miao et al. 2012). Microarray analysis revealed that transcriptional changes in 1816 genes were induced by *N*-hexanol-homoserine lactone (C6-HSL) (von Rad et al. 2008) and a total of 2873 genes were differentially expressed in response to *N*-3-oxo-hexanol-homoserine lactone (3OC6-HSL) (Zhao et al. 2016) in Arabidopsis. The majority of the differentially expressed genes could be assigned to the categories of energy metabolism, transcription/translation, biotic/abiotic stress adaptation, protein processing, signal transduction and hormone response (von Rad et al. 2008; Zhao et al. 2016). The short-chained AHLs such as C6-HSL, 3OC6-HSL and 3OC8-HSL can enhance primary root growth of Arabidopsis (von Rad et al. 2008; Jin et al. 2012; Liu et al. 2012; Zhao et al. 2015, 2016). Evidence suggested that G-protein, Ca^2+^/CaM signaling and transcriptional factor MYB44 are involved in AHL-induced primary root elongation (Liu et al. 2012; Song et al. 2011; Zhao et al. 2015, 2016). *N*-3-oxo-decanoyl-homoserine lactone (3OC10-HSL) influences the formation of adventitious roots via H_2_O_2_- and NO-dependent cGMP signaling in mung beans (Bai et al. 2012). *N*-decanoyl-homoserine lactone (C10-HSL) inhibited the primary root growth but stimulated the lateral root and root hair development (Ortiz-Castro et al. 2008). *N*-3-oxo-tetradecanoyl-Lhomoserine lactone (3OC14-HSL) significantly enhances the resistance against the bacterial pathogen *Pseudomonas syringae pv. tomato* DC3000 in Arabidopsis, and similar observations have been made in the case of biotrophic fungal pathogens (Schikora et al. 2011). Evidence suggested that mitogen-activated protein kinase AtMPK6, salicylic acid (SA) and oxylipin are required for AHL-induced resistance (Schikora et al. 2011; Shenk et al. 2014).

Salinity is a main environmental factor influencing plant growth and crop yield. High salt concentration in soil has a devastating effect on plant metabolism, disrupting cellular homeostasis and uncoupling major physiological and biochemical processes (Zou et al. 2019). Plants evolved a ubiquitous mechanism of salinity resistance, involving synthesis and accumulation of compatible compounds, sodium sequestration in vacuoles, enhancing antioxidant enzymes activities and so on (Zou et al. 2019). Salinity resistance can be further improved through the application of exogenous biostimulants (Bose et al. 2014). A plant biostimulant is a substance or microorganism that is beneficial to plants as growth promoters and stress protectors, regardless of its own nutrient content (Zou et al. 2019). Various biostimulants have been used in commercial agriculture, such as humic and fulvic acids, protein hydrolysates and other N-containing compounds, seaweed extracts and botanicals, chitosan and other biopolymers, inorganic compounds, beneficial fungi and bacteria (Du Jardin 2015). Rhizosphere bacteria can help plants tolerate abiotic stress, such as drought, salt, extreme temperature, nutrient deficiency and heavy metal toxicity (Dimkpa et al. 2009; Yang et al. 2009; Zhang et al. 2008). ‘Induced systemic tolerance’ (IST) was proposed for plant growth-promoting rhizobacteria (PGPR)-induced physical and chemical changes in plants that result in enhanced tolerance to abiotic stress (Yang et al. 2009). Inoculation of tomato plants with AHL-producing *Burkholderia graminis* M14 conferred dramatic protection against salt stress, and the transgenic tomato lines expressing *Yen*I (short-chain AHL producers) and *Las*I (long-chain AHL producers) exhibited salt tolerance (Barriuso et al. 2008). AHLs were considered as priming inducer to sensitize plant hosts to have the faster and the stronger responses to resist the subsequent stresses (Shenk and Schikora 2015). Proteomics analysis show that 23% of changed proteins induced by 3OC12-HSL and 3OC16:1-HSL related to defence response and stress response in M*edicago truncatula* (Mathesius et al. 2003). Microarray analysis show that 14.3% of the different expressing genes induced by 3OC6-HSL related to abiotic stress responses and the expression of 62 salt responsive genes were regulated in Arabidopsis seedlings (Zhao et al. 2016). However, little is known about the function of bacteria AHLs to the plant response to salt stress.

In present study, the effect of bacterial QS signal 3OC6-HSL on plant salt tolerance were analyzed in Arabidopsis and wheat. Shoot and root growth parameters, photosynthetic pigment, biochemical markers and ion contents were determined, and possible molecular mechanism of 3OC6-HSL-mediated salt tolerance was discussed in Arabidopsis and wheat.

## Methods

### Plant materials and growth conditions

*Arabidopsis thaliana* (L.) cv. Columbia-0 seeds were surface-sterilized by 75% (v/v) ethanol and 20% (v/v) NaClO and geminated on Murashige and Skoog (MS) Polygel medium (Murashige and Skoog 1962) adjusted to pH5.8. The seeds were stratified at 4 °C for 2 days and then grown in a growth chamber at 22 ± 2 °C and a 16 h light/8 h dark cycle with light intensity of 100 μmol m^2^ s^−1^. Sterile Hoagland medium were used for hydroponics and fertilized substrate TS1 (Klasmann-Deilmann GmbH, Germany) and vermiculite (1:2) were used for soil culture.

Wheat (*Triticum aestivum* L.) seeds were surface-sterilized by 75% (v/v) ethanol and soaked in water until germination, then transplanted into a floating hydroponic system containing sterile water at 25 ± 2 °C and under 16/8-h photoperiod (light/dark) with light intensity of 100 μmol m^2^ s^−1^.

### *N*-acyl-homoserine lactones (AHL) and salt stress treatments

The AHL, *N*-(β-ketocaproyl)-DL-homoserine lactone (3OC6-HSL), was purchased from Sigma-Aldrich (Deisenhofen, Germany), stored dry and diluted as 10 mM stock solutions in dH_2_O and sterilized by passing them through a 0.22-µm filter just prior to use. Solutions of 1 µM 3OC6-HSL or/and 150 mM NaCl were used as the treatments in our experiments. The untreated plants were taken as the control.

### Measurement of growth parameters

For primary root growth assay, Arabidopsis seeds were germinated vertically on MS Polygel plates for 3 days, then the seedlings with similar root length were selected to transfer to 1/2 MS Polygel plates containing 1 µM 3OC6-HSL or/and 150 mM NaCl. After cultured vertically for 6 days, primary root length was assessed using Image J software. The seeds of wheat were germinated and grown on sterile water containing 1 µM 3OC6-HSL or/and 150 mM NaCl for 6 days, then the root and shoot length were assessed using Image J software. The untreated plants were taken as the control. The experiments were replicated three times and each biological replication included at least 30 seedlings for each treatment.

For seedling-growth assay, 10 day-old seedlings of Arabidopsis on MS Polygel plates were transplanted into nutrition pots and 2 week-old soil-cultured seedlings were treated with 1 µM 3OC6-HSL or/and 150 mM NaCl. After 14 days, phenotypic changes of the seedlings were observed and the shoot length and fresh weight were measured. 2 week-old seedlings of wheat at the stage of two leaves and one shoot were treated with 1 µM 3OC6-HSL or/and 150 mM NaCl for 10 days, and the shoot length and fresh weight were measured. The untreated plants were taken as the control. The experiments were replicated four times and each biological replication included 15–30 seedlings for each treatment.

### Chlorophyll content

Chlorophyll content was determined according to Arnon (1949). 2 week-old Arabidopsis and wheat seedlings were treated with 1 µM 3OC6-HSL or/and 150 mM NaCl for 14 days in Arabidopsis and 10 days in wheat, and then harvested for chlorophyll determination. Untreated plants were used as controls. Total chlorophyll (Chl *a *+ *b*) were extracted from fresh leaf samples in 80% (v/v) acetone. The absorbance at 663 nm and 645 nm was measured using the UV–VIS spectrophotometer (model UV-2600, Shimadzu, Japan). The experiments were replicated three times and each biological replication included three replicated samples.

### Proline content

Proline content determination was performed as reported previously (Bates et al. 1973) with minor modification. 2 week-old Arabidopsis and wheat seedlings were treated with 1 µM 3OC6-HSL or/and 150 mM NaCl for 14 days in Arabidopsis and 10 days in wheat, and then harvested for proline determination. Untreated plants were used as controls. 100 mg fresh shoot samples were homogenized in 1.5 ml of 0.3% (w/v) sulphosalicylic acid, then centrifuged at 10,000×*g* for 10 min. 100 μl of supernatant was reacted with 1 ml glacial acetic acid and 1 ml acid ninhydrin at 100 °C for 30 min, and cooled on ice to terminate the reaction. Then the reaction mixture was extracted with 1 ml toluene and the absorbance at 520 nm were measured using the UV–VIS spectrophotometer (model UV-2600, Shimadzu, Japan). Proline concentration was determined by the proline standard curve. The experiments were replicated three times and each biological replication included three replicated samples.

### Lipid peroxidation

Amount of lipid peroxidation was determined by estimating the malondialdehyde (MDA) produced by the thiobarbituric acid (TBA) reaction according to Del Buono et al. (2011) with minor modification. 2 week-old Arabidopsis and wheat seedlings were treated with 1 µM 3OC6-HSL or/and 150 mM NaCl for 14 days in Arabidopsis and 10 days in wheat, and then harvested for MDA determination. Untreated plants were used as controls. 100 mg fresh shoot samples were homogenized with 10% (w/v) TCA and centrifuged at 4000×*g* for 10 min. 1 ml of 0.6% (w/v) TBA was added to 1 ml of supernatant. The mixture was heated at 100 °C for 15 min and immediately cooled on ice, then centrifuged at 10,000×*g* for 15 min. Absorbance at 450, 532 and 600 nm were measured using the UV–VIS spectrophotometer (model UV-2600, Shimadzu, Japan). The experiments were replicated three times and each biological replication included three replicated samples.

### Measurement of Na^+^ and K^+^ concentrations

Atomic absorption was used to determine the Na^**+**^ and K^**+**^ content, as conducted by Zhao et al. (2009). 2 week-old Arabidopsis and wheat seedlings were treated with 1 µM 3OC6-HSL or/and 150 mM NaCl for 14 days in Arabidopsis and 10 days in wheat, and then harvested for ion determination. Untreated plants were used as controls. Shoot samples were dried at 80 °C for 48 h. 50 mg dry samples were digested with 3 ml of nitric acid, and Na^**+**^ and K^**+**^ concentrations were determined with an atomic absorption spectrometer (model A240FS + 240Z; Varian, Palo Alto, CA). The experiments were replicated three times and each biological replication included three replicated samples.

### Quantitative Real-Time (qRT) PCR analysis

20 day-old hydroponic cultured Arabidopsis seedlings were treated with 1 µM 3OC6-HSL or/and 150 mM NaCl for 6 h and then harvested for RNA extraction. Untreated plants were used as controls. Total RNA was extracted using the RNAiso Plus reagent (TaKaRa, Dalian, China). cDNA was synthesized using the PrimeScript^®^ RT Reagent Kit with gDNA Eraser (TaKaRa, Dalian, China) according to the manufacturer’s instructions. SYBR Premix Ex Taq was purchased from TaKaRa (Shiga, Japan). For the relative quantification of gene expression, the comparative C_T_ method (Livak and Schmittgen 2001) was used with the 7500 Real Time PCR System (Applied Biosystems, Foster City, CA, USA). The amount of target was normalized to the endogenous reference gene *ACTIN2*. For technical control, each sample was repeated four times on the same 96-well plate. Each data point represents the average of three independent experiments. A 1.5-fold increase (ratio > 1.5) or 1.5-fold decrease (ratio < 0.8) in expression in the treated plants compared with the untreated plants (the control) was considered as up-regulation or down-regulation related to stress response. The specific primers that we used for qRT-PCR are shown in Additional file 1: Table S1.

### Statistical analysis

For all experiments, the overall data were statistically analyzed in the DPS v7.05 program. Univariate and multivariate analyses (ANOVA) with a Duncan’s new multiple range tests (*P *< 0.05) were used. All data were represented as mean ± SD of three or four independent experiments.

## Results

### 3OC6-HSL is helpful to plant growth under salt stress

**Fig. 1:**
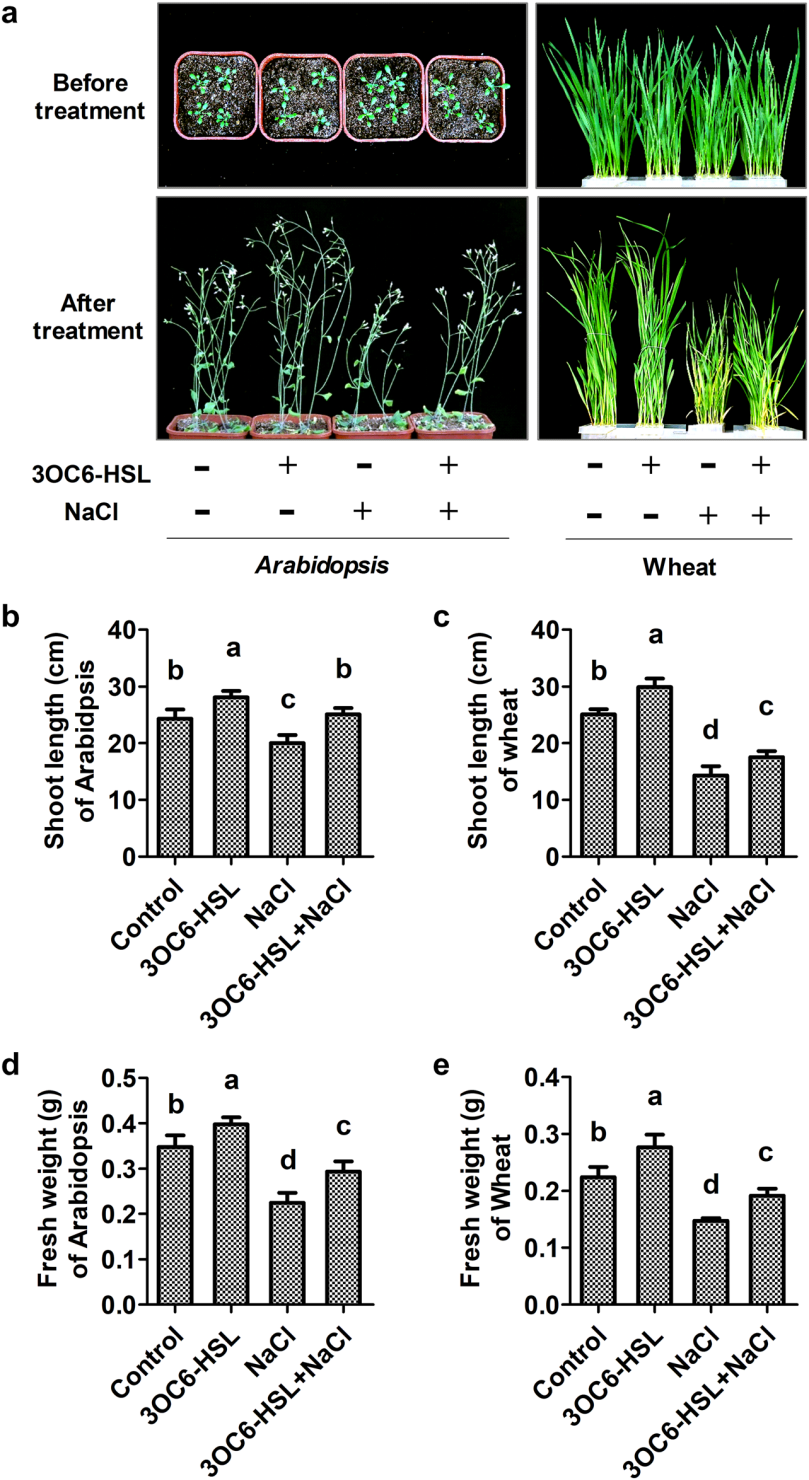
Effect of 3OC6-HSL on plant growth in Arabidopsis and wheat. 2-week-old seedlings were treated with 1 μM 3OC6-HSL or/and 150 mM NaCl as indications. The changes of plant growth phenotype were observed after treatment for 14 days in Arabidopsis and 10 days in wheat. **a** Plant growth status; **b** shoot length of Arabidopsis; **c** fresh weight of Arabidopsis; **d** shoot length of wheat; **e** fresh weight of wheat. Values are mean ± SD of four independent experiments. Different letters indicate statistically significant differences (*P* < 0.05, Duncan’s test)

The influence of bacterial QS signal 3OC6-HSL on seedling growth of Arabidopsis and wheat were analyzed in both saline and non-saline environment. Application of 3OC6-HSL can significantly enhance plant growth in both Arabidopsis and wheat under non-saline condition (Fig. 1a). Compared with the untreated control, the shoot length increased by 15.6% and 19.2% (Fig. 1b, d), and the fresh weight increased by 14.4% and 23.7% (Fig. 1c, e) in 3OC6-HSL-treated Arabidopsis and wheat, respectively. Under salt stress condition, the phenotype of dwarf and yellow wilt caused by high salinity were significantly improved by 3OC6-HSL in both Arabidopsis and wheat (Fig. 1a). Compared with the NaCl-treated plants, the shoot length increased by 25.5% and 22.4% (Fig. 1b, d) and the fresh weight increased by 30.6% and 29.9% (Fig. 1c, e) in 3OC6-HSL and NaCl co-treated Arabidopsis and wheats, respectively. These results indicated that 3OC6-HSL played an important role in plant growth under salt stress.

### 3OC6-HSL promotes plant root elongation under salt stress

**Fig. 2:**
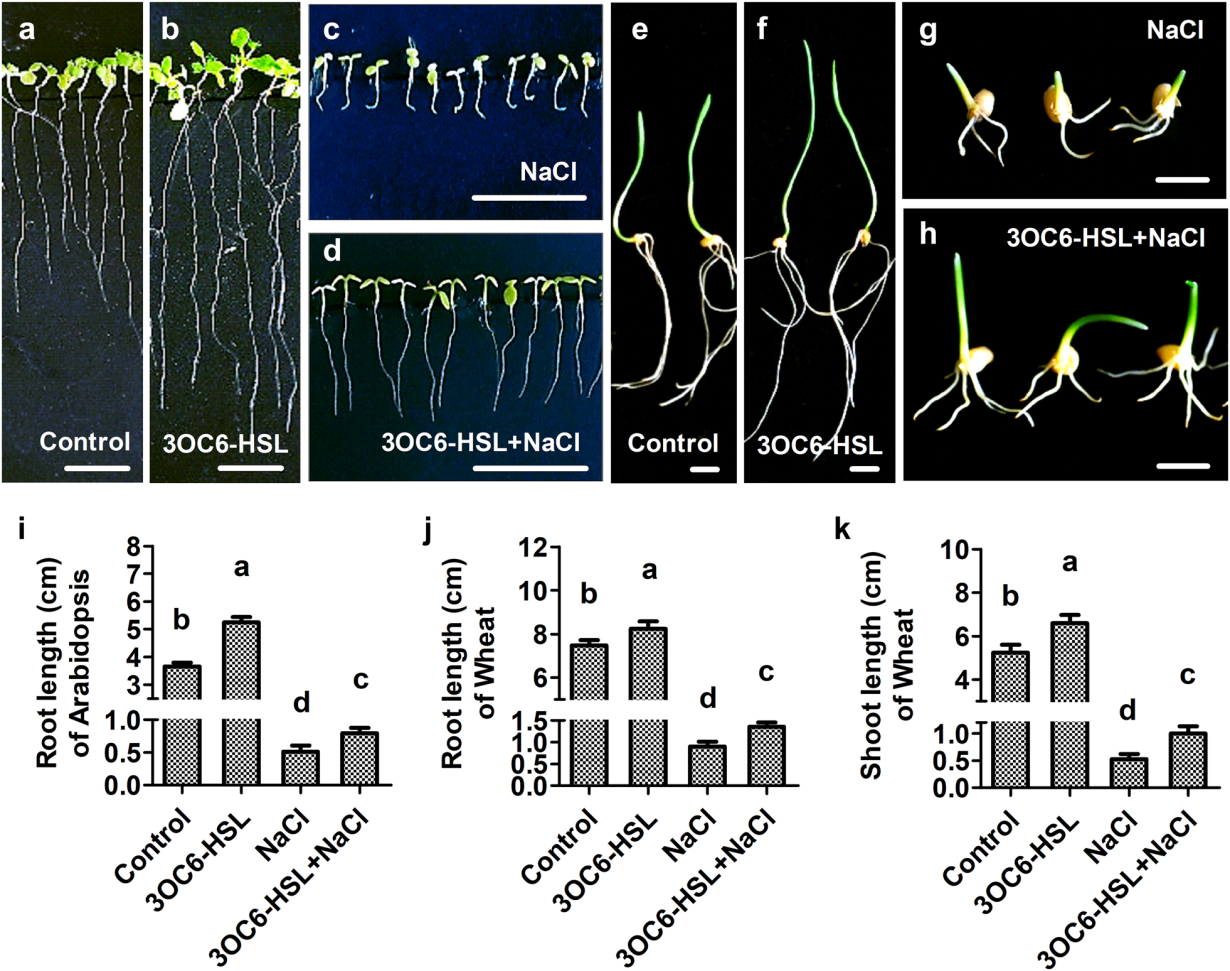
Effect of 3OC6-HSL on root growth in Arabidopsis and wheat. Seedlings were treated with 1 μM 3OC6-HSL or/and 150 mM NaCl after seeds germination. The changes of root phenotype were observed after 6 days treatment in Arabidopsis and wheat. **a**, **e** Non-treated control plants; **b**, **f** 3OC6-HSL treated plants; **c**, **g** NaCl treated plants; **d**, **h** 3OC6-HSL and NaCl co-treated plants; **i** root length of Arabidopsis; **j** root length of wheat; **k** shoot length of wheat. bar = 1 cm, values are mean ± SD of three independent experiments. Different letters indicate statistically significant differences (*P* < 0.05, Duncan’s test)

The effect of 3OC6-HSL on root growth of Arabidopsis and wheat were analyzed in both saline and non-saline environment. 3OC6-HSL induced plant root elongation significantly under non-saline condition (Fig. 2a, b, e, f) and relieved root growth inhibition significantly under salt stress condition (Fig. 2c, d, g, h) in both Arabidopsis and wheat. Compared with the non-3OC6-HSL-treated plants, the primary root length increased by 43.7% and 55.1% in 3OC6-HSL-treated Arabidopsis under non-saline and saline condition, respectively (Fig. 2i). Similarly, the root length increased by 10.1% and 50.1% and the shoot length increased by 25.8% and 89.9% in 3OC6-HSL-treated wheat under non-saline and saline condition, respectively, compared with those of non-3OC6-HSL-treated plants (Fig. 2j, k). These results indicated that 3OC6-HSL played a positive role in plant early growth and root elongation under salt stress.

### 3OC6-HSL influents biochemical markers related to plant salt tolerance

The effect of 3OC6-HSL on biochemical markers related to plant salt tolerance, including chlorophyll, proline and MDA, were analyzed in Arabidopsis and wheat under both saline and non-saline condition.

**Fig. 3:**
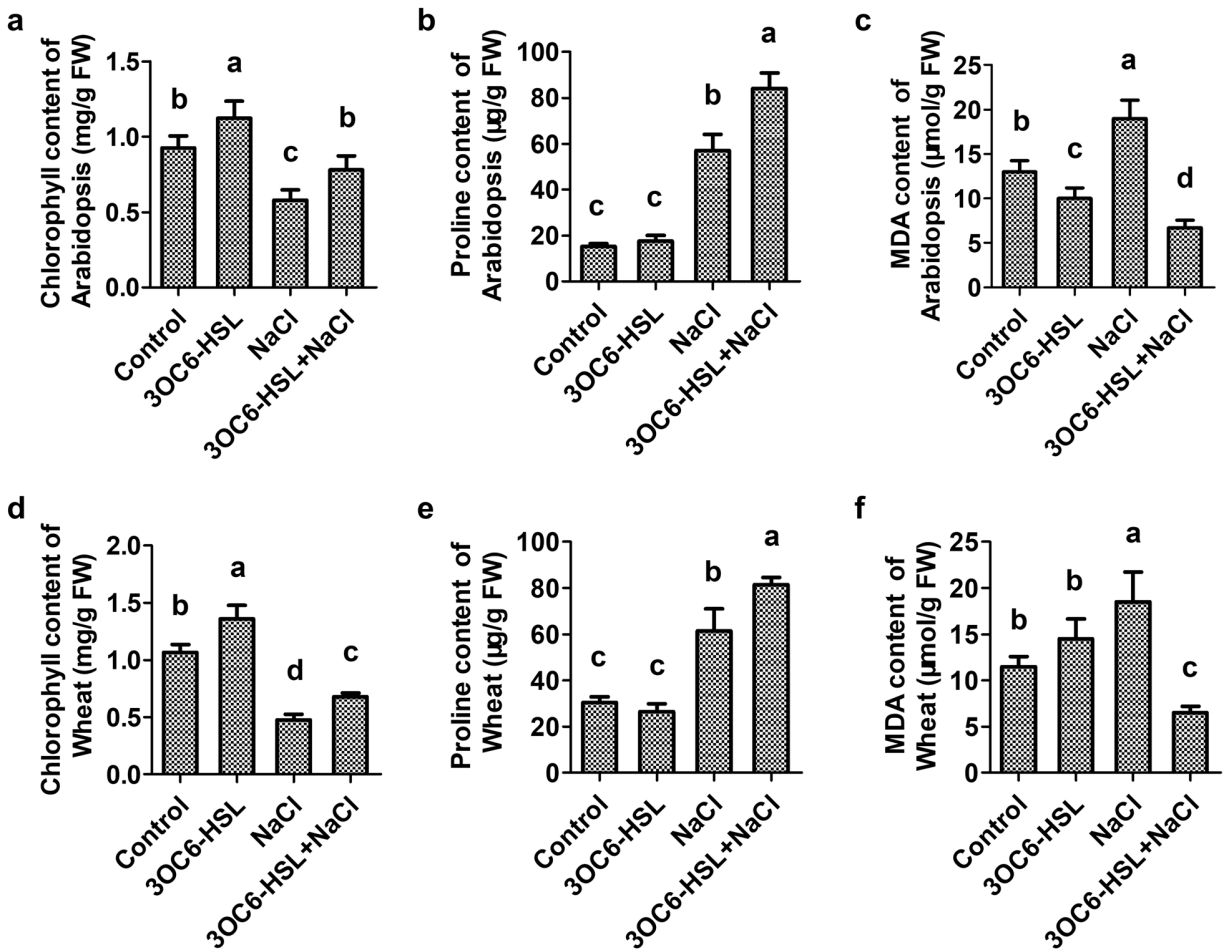
Effect of 3OC6-HSL on biochemical markers related to salt toleranc in Arabidopsis and wheat. 2-week-old seedlings were treated with 1 μM 3OC6-HSL or/and 150 mM NaCl as indications. The levels of total chorophyll, proline and MDA were measured after treatment for 14 days in Arabidopsis (**a**–**c**) and 10 days in wheat (**d**–**f**). Values are mean ± SD of three independent experiments. Different letters indicate statistically significant differences (*P* < 0.05, Duncan’s test)

Chlorophyll level is widely used as an index of abiotic stress tolerance in plant. Compared with the control plants, total chlorophyll (chla + chlb) content increased by 21.1% and 27.3% in 3OC6-HSL-treated Arabidopsis and wheat, respectively (Fig. 3a, d). And compared with NaCl-treated plants, total chlorophyll content increased by 34.9% and 43.1% in 3OC6-HSL and NaCl co-treated Arabidopsis and wheat, respectively (Fig. 3a, d). 3OC6-HSL induced a significant raise of total chlorophyll content under non-saline condition and relieved significantly the decrease of total chlorophyll content caused by high salinity in both Arabidopsis and wheat.

Accumulation of osmolyte proline facilitates plant to overcome abiotic stress by maintaining osmotic turgor (Tiwari et al. 2017). There were no significant changes in proline content among 3OC6-HSL-treated and non-3OC6-HSL-treated Arabidopsis and wheat under non-saline condition, whereas, an increase was registered when plants were exposed in salt environment (Fig. 3b, e). However, a higher level of proline was induced by 3OC6-HSL under salt stress condition, which were increased by 47.4% and 32.6% relative to the control in Arabidopsis and wheat, respectively (Fig. 3b, e).

MDA is an indicator of extent of lipid peroxidation and membrane damage in plant during salt stress. Decline of MDA content was observed in Arabidopsis with 3OC6-HSL treatment, especially under salt stress condition. The ratio of MDA decline was 23.1% and 64.7% under non-saline condition and saline condition, respectively, in comparison to the non-3OC6-HSL-treated Arabidopsis (Fig. 3c). Compared with the non-3OC6-HSL-treated wheat, a slight higher MDA content (26.1% of increase) was shown in 3OC6-HSL-treated wheat under normal condition while significant lower MDA content (64.9% of decrease) was shown in 3OC6-HSL-treated wheat under salt stress condition (Fig. 3f).

### 3OC6-HSL is helpful to modulate the level of cellular Na^+^ under salt stress

**Fig. 4:**
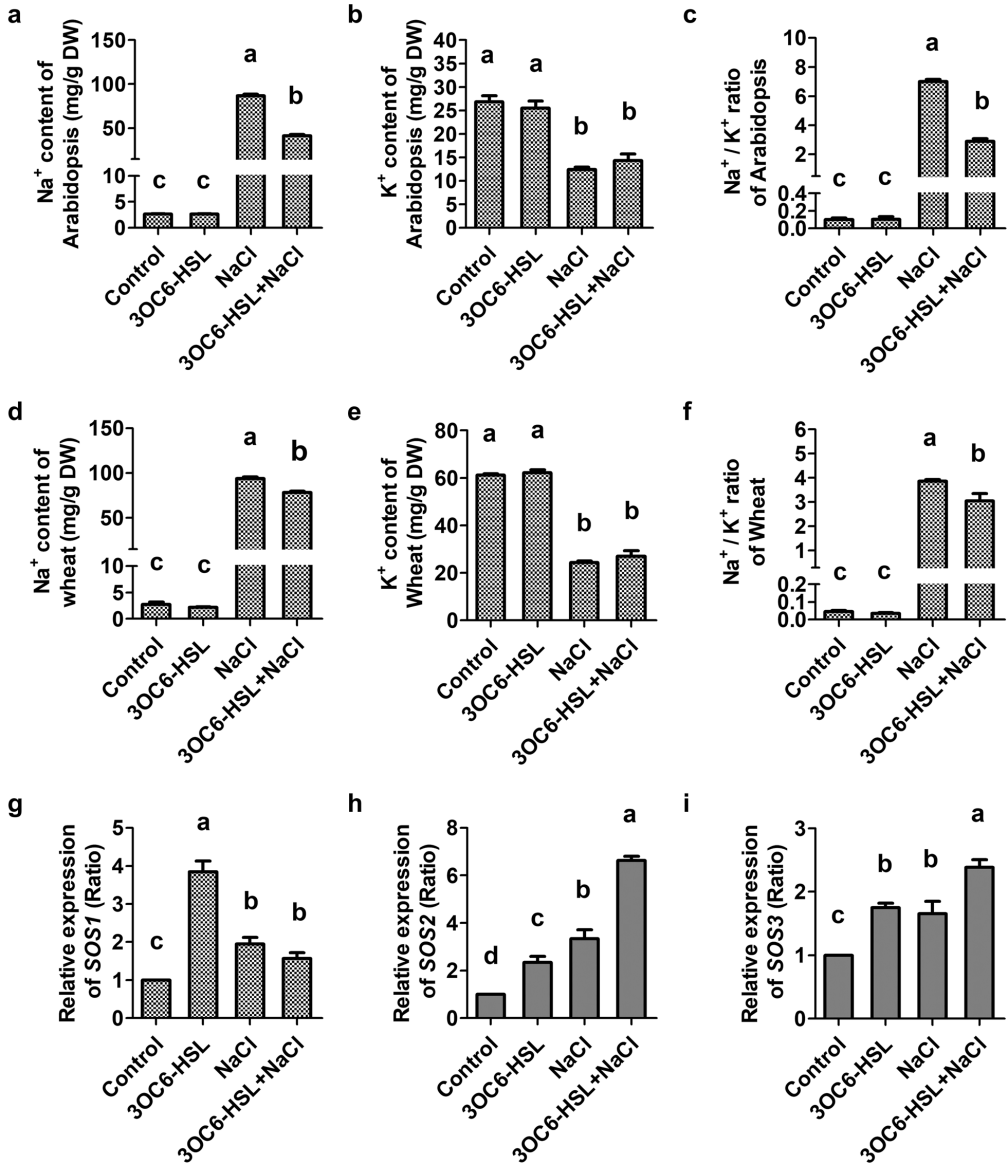
Effect of 3OC6-HSL on cellular ionic homeostasis in Arabidopsis and wheat. 2-week-old seedlings were treated with 1 μM 3OC6-HSL or/and 150 mM NaCl as indications. The contents of Na^+^ and K^+^ were measured and Na^+^/K^+^ ratio were calculated after treatment for 14 days in Arabidopsis (**a**–**c**) and 10 days in wheat (**d**–**f**). 20-day-old seedlings were treated with 1 μM 3OC6-HSL or/and 150 mM NaCl as indications. The relative gene expression of *SOS1* (**g**), *SOS2* (**h**) and *SOS3* (**i**) were measured by qRT-PCR after treatment for 6 h in Arabidopsis. Values are mean ± SD of three independent experiments. Different letters indicate statistically significant differences (*P* < 0.05, Duncan’s test)

The effect of 3OC6-HSL on cellular ion contents were analyzed in Arabidopsis and wheat under both saline and non-saline condition. There was no significant difference of Na^+^ and K^+^ contents between the control and 3OC6-HSL-treated Arabidopsis and wheat under non-saline condition (Fig. 4a, b, d, e). High salinity led to cellular Na^+^ content increase sharply, while K^+^ content decline. However, high Na^+^ contents reduced significantly with 3OC6-HSL application in Arabidopsis and wheat under salt stress condition. The level of cellular Na^+^ decreased by 52.4% and 16.7% in 3OC6-HSL and NaCl co-treated Arabidopsis and wheat, respectively, compared with NaCl-treated seedlings (Fig. 4a, d). There were no change in K^+^ content after 3OC6-HSL treatment in Arabidopsis and wheat under salt stress condition (Fig. 4b, e). Consequently, lower Na^+^/K^+^ ratio was observed in 3OC6-HSL and NaCl co-treated Arabidopsis and wheat under saline condition (Fig. 4c, f).

For verifying the effect of 3OC6-HSL on cellular ionic homeostasis, the transcriptional expression level of Salt Overly Sensitive (SOS) pathway genes, including *SOS1*, *SOS2* and *SOS3* was analyzed in Arabidopsis under both saline and non-saline condition. The expression of *SOS1*, *SOS2* and *SOS3* was up-regulated by 3OC6-HSL to 3.8-, 2.3- and 1.8-fold, respectively, compared with untreated control, under non-saline condition (Fig. 4g–i). There was higher expression level of *SOS2* and *SOS3* in 3OC6-HSL-treated seedling than in non-3OC6-HSL-treated seedling under salt stress condition (Fig. 4h, i). Although no significant change of *SOS1* expression level was observed between NaCl-treated seedling and 3OC6-HSL and NaCl co-treated seedling, 3OC6-HSL could strongly induce *SOS1* expression under normal condition (Fig. 4g).

### 3OC6-HSL upregulated the transcriptional expression of both ABA-dependent and ABA-independent salt related genes

**Fig. 5:**
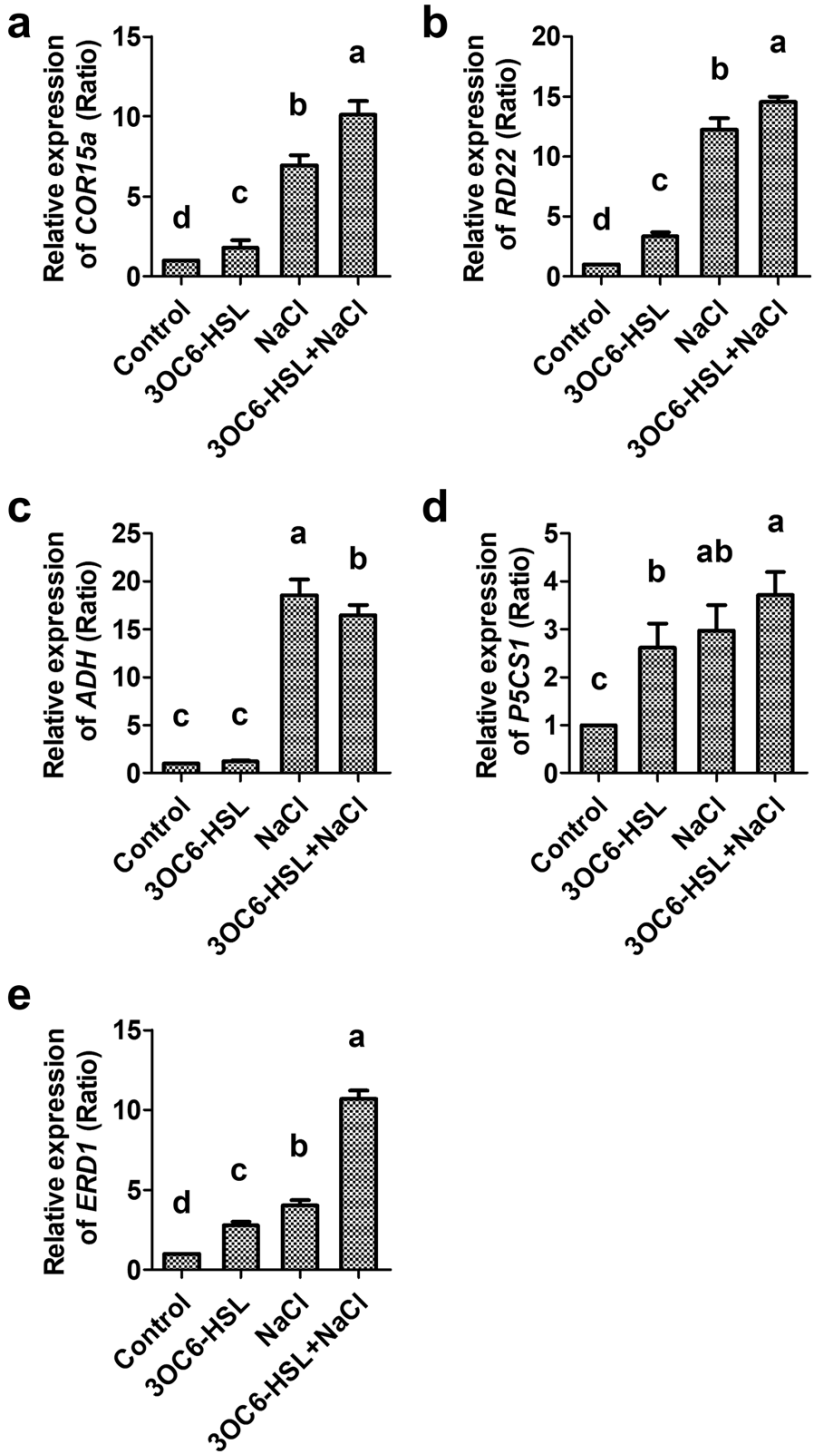
Effect of 3OC6-HSL on the expression of ABA-dependent and ABA-independent genes in Arabidopsis. 20-day-old seedlings were treated with 1 μM 3OC6-HSL or/and 150 mM NaCl as indications. The relative gene expression of *COR15a* (**a**), *RD22* (**b**), *ADH* (**c**), *P5CS1* (**d**) and *ERD1* (**e**) were measured by qRT-PCR after treatment for 6 h in Arabidopsis. Values are mean ± SD of three independent experiments. Different letters indicate statistically significant differences (*P* < 0.05, Duncan’s test)

*COR15a*, *RD22*, *ADH* and *P5CS1* genes belonged to ABA-dependent genes in plant salt signaling pathway (Xiong et al. 2001). The expression of *COR15a*, *RD22* and *P5CS1* was up-regulated by 3OC6-HSL to 1.8-, 3.4- and 2.6-fold under non-saline condition, respectively, compared with untreated control (Fig. 5a, b, d). There was higher expression level of *COR15a* and *RD22* in 3OC6-HSL-treated seedling than in non-3OC6-HSL-treated seedling under salt stress condition (Fig. 5a, b). Although no significant difference of *P5CS1* expression level was observed between NaCl-treated seedling and 3OC6-HSL and NaCl co-treated seedling, 3OC6-HSL could strongly induce *P5CS1* expression under normal condition (Fig. 5d). The expression of *ADH* was not up-regulated by 3OC6-HSL though it could be strongly induced by NaCl (Fig. 5c).

*ERD1* represents ABA-independent gene in plant salt signaling pathway (Nakashima et al. 1997). 3OC6-HSL induced an increased expression of *ERD1* by 2.8-fold compared with untreated control under non-saline condition (Fig. 5e). Under saline condition, the expression of *ERD1* was up-regulated significantly by NaCl and higher expression (2.6-fold compared with NaCl-treated sample) was induced by 3OC6-HSL (Fig. 5e).

## Discussion

Salinity is one of the major abiotic stressors that impairs plant growth and development, for instance, reduction of cell elongation and cell division rates, younger leaves emerge slowly, lateral buds remain quiescent, leaf senescence and flowering starts earlier (Passioura and Munns 2000; Ilangumaran and Smith 2017). Salt tolerance is usually quantified over a given period as survival, vegetative growth, or harvestable biomass at different physiological stages of the plant in saline versus non-saline conditions (Munns 2002). Bacterial QS signal AHLs affect the growth and modulate root structures of dicot and monocot plants. C6-HSL, 3OC6-HSL and 3OC8-HSL promote primary root elongation of Arabidopsis (von Rad et al. 2008; Liu et al. 2012; Zhao et al. 2015). C6-HSL, C8-HSL and C12-HSL promote shoot growth and induce lateral root formation in barley (Klein et al. 2009; Rankl et al. 2016). 3OC10-HSL influences the formation of adventitious roots in mung beans (Bai et al. 2012). Growth promoting effect of AHLs is helpful for plant to withstand environmental stimuli stresses. In present study, effect of 3OC6-HSL on salt tolerance was analyzed in the early and middle vegetative growth period in Arabidopsis and wheat. Application of 3OC6-HSL can significantly increase the shoot length, root length and fresh weight in Arabidopsis and wheat under both saline and non-saline conditions (Figs. 1b–e and 2i–k). The growth status of dwarf and yellow wilt caused by high salinity were significantly improved by 3OC6-HSL treatment (Figs. 1a and 2a–h). Thus, 3OC6-HSL appears to be a potent biostimulator to enhance salt tolerance of Arabidopsis and wheat mainly because of its effect on growth promotion.

Salinity restricts plant growth and development by influencing physiological and biochemical processes. Abiotic stresses seriously affect photosynthesis in plant, such as reduction in chlorophyll content, disintegration of chloroplast membranes and disruption of photosystem biochemical reactions (Zou et al. 2019). To increase chlorophyll content is helpful to improve plant growth in saline environment and enhance plant salt tolerance (Zou et al. 2019). Degradation of chlorophyll under abiotic stress is usually related to the accumulation of ROS, which leads to lipid peroxidation of chloroplast membranes (Zou et al. 2019). MDA is an indicator of extent of lipid peroxidation and membrane damage in plant (Lata et al. 2011). In present study, 3OC6-HSL significantly increased chlorophyll content and decreased MDA content in Arabidopsis and wheat under both saline and non-saline condition (Fig. 3a, d). These results indicated that 3OC6-HSL played an important role in enhancing photosynthetic efficiency of plant and protecting plant from membrane destruction.

Plant salt stress can be divided into early-occurring osmotic stress stage and slowly increasing ionic stress stage (Sahi et al. 2006; Munns and Tester 2008). The accumulation of organic osmolytes such as proline, glycine betaine, suger alcohols, polyamines and so on, plays a key role in maintaining the low intracellular osmotic potential of plants and preventing the harmful effects of salt stress (Verslues et al. 2006). Proline not only plays a crucial role in osmotic adjustment but also acts as a reactive oxygen scavenger, redox buffer, or molecular chaperone, stabilizing proteins and membrane structures under stress conditions (Ashraf and Foolad 2007; Verbruggen and Hermans 2008). Proline also induced the expression of salt stress-responsive genes, which promoted the adaptation of plants to salt stress (Zhang et al. 2017). In Arabidopsis, knockout of the *P5CS1* gene, which encodes a 1-pyrroline-5-carboxylate synthetase that central for proline biosynthesis, impairs proline synthesis resulting in salt hypersensitivity (Székely et al. 2008). In present study, 3OC6-HSL increased the content of proline significantly and up-regulated the expression of *P5CS1* in Arabidopsis and wheat under non-saline and saline conditions, though there was no significant difference in the expression of *P5CS1* between NaCl treated and 3OC6-HSL and NaCl co-treated seedlings under salt stress (Fig. 3b, e). These results indicated that 3OC6-HSL can activate proline biosynthesis and osmotic modulation in plant under salt stress.

Plant resistance to osmotic stress can be regulated through ABA-dependent and ABA-independent signaling pathways (Bharti et al. 2016). Besides *P5CS1*, ABA-dependent osmotic regulation marker genes including *COR15a*, *RD22* and *ADH*, and ABA-independent marker gene *ERD1* were investigated in Arabidopsis and wheat with or without 3OC6-HSL treatment under saline and non-saline condition. *COR15a* belongs to late embryogenesis abundant protein (LEA) family and is important for plant tolerance to freezing-induced cellular dehydration (Sowemimo et al. 2019). *COR15a* contains ABA responsive element (ABRE) in several plant species (Kim 2006) and can be induced by ABA, cold and salinity (Zhu et al. 2017; Sowemimo et al. 2019). The promotor of *RD22* can be recognized by MYB/MYC transcription factors, which are induced by ABA, drought and salinity (Abe et al. 2003). Early Responsive to Dehydration Stress 1 (*ERD1*) encodes a ClpA (ATP binding subunit of the caseinolytic ATP-dependent protease) homologous protein and its promotor contains drought-responsive cis-element (DRE) that activated by NAC transcription factors under drought and salt stress (Tran et al. 2004). In present study, the expression of *COR15a*, *RD22* and *ERD1* were up-regulated by 3OC6-HSL in Arabidopsis and wheat under saline and non-saline condition (Fig. 5a, b, e). Furthermore, significant higher expression level of *COR15a*, *RD22* and *ERD1* in 3OC6-HSL-treated seedling was observed than in non-3OC6-HSL-treated seedling under salt stress condition (Fig. 5a, b, e). These result implicated that 3OC6-HSL regulated plant salt tolerance through not only ABA-dependent pathway but also ABA-independent pathway.

Plants minimize the harmful effects of ionic Na^+^ stress by exclusion of Na^+^ from leaf tissues and compartmentalization of Na^+^ into vacuoles (Munns and Tester 2008; Blumwald 2000). SOS signaling pathway is a key mechanism for exclusion of Na^+^ and ion homeostasis control at cellular level (Zhu 2000). SOS3, a calcium binding protein, is responsible for sensing calcium signals caused by salinity, and then dimerized and activated SOS2 serine/threonine protein kinase. SOS2/SOS3 complex phosphorylated plasma membrane Na^+^/H^+^ antiporter SOS1 resulting in Na^+^ efflux (Zhu 2002; Munns and Tester 2008). SOS1 also regulates the transportation of Na^+^ from root to shoot to maintain appropriate K^+^/Na^+^ ratio in leaves (Qiu et al. 2002). Mutation studies show that SOS2/SOS3 complex is involved in negative regulation of AtHKT1 (Isayenkov and Maathuis 2019), which is responsible for Na^+^ translocation to the shoot (Halfer et al. 2000). SOS2 can also interact with vacuolar Na^+^/H^+^ exchanger (NHX) antiporters and significantly elevate their exchange activity (Zhu 2002). In present study, the expression of *SOS1*, *SOS2* and *SOS3* were strong induced by 3OC6-HSL in Arabidopsis under both saline and non-saline condition (Fig. 4g–i). What’s more, significant higher expression level of *SOS2* and *SOS3* in 3OC6-HSL-treated seedling was observed than in non-3OC6-HSL-treated seedling under salt stress condition (Fig. 4h, i). Meanwhile, 3OC6-HSL reduced Na^+^ contents significantly and maintained lower cytosolic Na^+^/K^+^ ratio in Arabidopsis and wheat under salt stress condition (Fig. 4a, c, d, f). These results indicated that 3OC6-HSL could modulate cytosolic ion homeostasis by regulating SOS signal pathway under salt stress.

In summary, bacterial 3OC6-HSL can enhance plant salt tolerance. ABA-dependent and ABA-independent signal pathways and SOS signaling pathway might participate the regulation of salt tolerance by 3OC6-HSL in plants. It provides a new insight into the plant–microbe inter-communication. Salt stress in plant is a cumulative effect of osmotic and ionic stress which negative affects the plant growth and yield and multiple genes are involved in salt tolerance mechanism (Bharti et al. 2016). Further study should be conducted to clarify the molecular mechanism of a bacterial bioactive compound to improve plant growth and stress resistance. Molecular breeding and advanced biotechnology methods should help scientists to develop crops with enhanced salt tolerance (Deinlein et al. 2014). An AHL binding-Fe-CNF nanocomposite (NC) was successfully used as a nano-biofertilizer for stimulating seed germination and seedling growth, and developing resistance to oxidative and salinity stress and to fungal pathogenesis in chickpea (Gupta et al. 2019). Bacterial 3OC6-HSL is hopeful to apply as a biostimulant to promote crops growth and protect crops from salinity injury in agriculture production.

## Supplementary information

**Additional file 1: Table S1.** Primer information of genes investigated in qRT-PCR.

## Data Availability

Not applicable
