# Supplementary material for: N-3-oxo-hexanoyl-homoserine lactone, a bacterial quorum sensing signal, enhances salt tolerance in Arabidopsis and wheat
Source: Bot Stud. 2020 Mar 10;61:8. doi: 10.1186/s40529-020-00283-5 (PMC7064656; doi:10.1186/s40529-020-00283-5)
Supplement: Supplementary file 1 — Additional file 1: Table S1. Primer information of genes investigated in qRT-PCR. [file 40529_2020_283_MOESM1_ESM.pdf]

Additional File 1: Table S1. Primer information of genes investigated in qRT-PCR

Gene	Forward Primer (5’→3’)	Reverse Primer (5’→3’)
ERD1	ATTGCTGCTATGCTTTTCTGTGGAC	CGAACAAAACCACTGTAAAAGGACG
RD29B	ATGAGTATGACGAGCAAGACCCAGA	TGTGGTCAGAAGACACGACAGGAA
RD22	TACCAAACACTCCCATTCCCAACT	CACCTCCGTGACCTTTTCCG
COR15a	TTTCCACAGCGGAGCCAAG	ACCATCTGCTAATGCCTCTTTTGT
P5CS1	TGTTCCACAACGCCAGCAC	TGGGAATGTCCTGATGGGTG
ADH	CTCTTGGTGCTGTTGGTTTAGG	AATTGGCTTGTCATGGTCTTTC
SOS1	TTCATCATCCTCACAATGGCTCTAA	TATTCTGCCCTCATCAAGCATCTCC
SOS2	TATTTGGTCTTGCGGGGTTAT	GATTGGGGTCAAGTATCCTATGTAT
SOS3	CGGTCCTTAGGTGTCTTCCATCC	GCACGAAAGCCTTATCCACCAT
Actin2	CCAGAAGGATGCATATGTTGGTGA	GAGGAGCCTCGGTAAGAAGA
